# Analgesic and Anti-Inflammatory Activities of Resveratrol through Classic Models in Mice and Rats

**DOI:** 10.1155/2017/5197567

**Published:** 2017-03-13

**Authors:** Guangxi Wang, Zhiqiang Hu, Xu Song, Qiankun Cui, Qiuting Fu, Renyong Jia, Yuanfeng Zou, Lixia Li, Zhongqiong Yin

**Affiliations:** ^1^Natural Medicine Research Center, College of Veterinary Medicine, Sichuan Agricultural University, Chengdu 611130, China; ^2^Key Laboratory of Animal Disease and Human Health of Sichuan Province, Sichuan Agricultural University, Chengdu 611130, China

## Abstract

*Background*. Inflammation and pain are closely related to humans' and animals' health.* Resveratrol* (RSV) is a natural compound with various biological activities. The current study is aimed to evaluate the analgesic and anti-inflammatory activities of RSV in vivo*. Materials and Methods*. The analgesic effects were assessed by the acetic acid-induced writhing and hot plate tests. The anti-inflammatory effects were determined using the xylene-induced mouse ear oedema, the acetic acid-induced rat pleurisy, and carrageenan-induced rat synovitis tests, respectively.* Results*. The analgesic results showed that RSV could significantly inhibit the number of writhes and improve the time and pain threshold of mice standing on hot plate. The anti-inflammatory results showed that RSV could inhibit the ear oedema of mice. In acetic acid-induced pleurisy test, RSV could significantly inhibit the WBC and pleurisy exudates, could decrease the production of NO, and elevate the activity of SOD in serum. In carrageenan-induced synovitis test, RSV could reduce the content of MDA and elevate the T-SOD activity in serum; RSV could inhibit the expressions of TP, PGE2, NO, and MDA.* Conclusion*. Shortly, these results indicated that RSV had potent analgesic and anti-inflammatory activities and could be a potential new drug candidate for the treatment of inflammation and pain.

## 1. Introduction

Pain and inflammation are implicated in virtually all human and animal diseases, and they are usually produced by physical, chemical, and biological stimuli, or some combination of these [1]. The typical characteristics of inflammation are redness, swelling, heat, pain, and dysfunction. Therefore, there are always interactions between pain and inflammation. Analgesics are a kind of medicines in general which can relieve the feeling of pain. Conventional analgesics play an important role in pain therapy, but they always cause kinds of adverse effects during clinic use [2]. The same as the analgesics, nonsteroidal anti-inflammatory drugs (NSAIDs) are the primary therapy for diseases with a chronic inflammatory response, but long-term use often causes severe side effects, including cardiovascular and gastrointestinal complications that limit their development [3, 4]. Therefore, the research for new analgesic and anti-inflammation agents are critically needed.

In the last few decades, people had discovered many plants with analgesic property and numerous herbal preparations are being suggested as analgesics [5]. Meanwhile, due to the wide range of pharmacological activities with less side effects, there are many reports about anti-inflammatory activities of components from Chinese traditional medicine, including* alkaloids, saponins, flavonoids, terpenoids, volatile oils, coumarin, aldehydes, *and* ketones* [6, 7].

**Figure 1:**
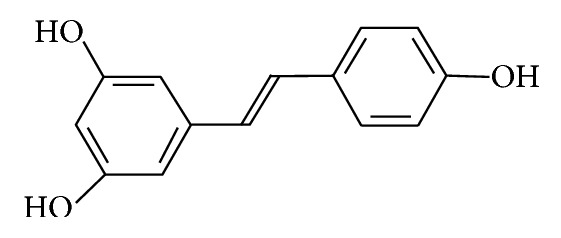
Chemical structure of trans-resveratrol.

*Resveratrol* (RSV), 3,4′,5-trihydroxy-trans-stilbene, is a stilbene-type aromatic phytoalexin mainly found in grapes, peanuts, berries, turmeric, and other food products [8]. Two different RSV structures exist (cis-RSV and trans-RSV), but we usually refer to its trans-structure (Figure 1). Since RSV becomes the focal point of human, there are many reports about RSV that exhibits several physiological activities, including anticancer, antioxidant, and anti-inflammatory both in vitro and in vivo [9–11]. Though the anti-inflammatory effect of RSV has been widely researched and even goes deep into its mechanism [11–13], there were no systematic studies for RSV on inflammatory diseases and too little attention has been paid to its analgesic activity. Hence, this study is conducted to systematically investigate the analgesic and anti-inflammatory activities of RSV for the purpose of developing a new drug for the treatment of inflammation and pain.

## 2. Materials and Methods

### 2.1. Drugs and Reagents

RSV (98%) was bought from Xian SenZhuo Technology Co., Ltd. (Xian, China). Acetic acid (AC) was bought from Wuhan Jiangbei Chemical Reagent Co., Ltd. (Wuhan, China). Indomethacin was bought from Shanxi SanJin Pharmaceutical Co., Ltd. (Shanxi, China). Xylene was bought from Hubei Shashi Chemical Reagent Co., Ltd. (Hubei, China). Dexamethasone (DXM) was bought from Tianjin Pharmaceutical Group Co., Ltd. (Tianjin, China). Carrageen was bought from the Sigma Company (USA); nitrogen monoxide (NO), malondialdehyde (MDA), superoxide dismutase (SOD), total superoxide dismutase (T-SOD), prostaglandin E2 (PEG_2_), and total protein (TP) kits were all bought from Nanjing Jiancheng Bioengineering Institute (Nanjing, China).

### 2.2. Animal Preparation

Young adult males (average weight 18 ± 2 g) and females (average weight 18 ± 2 g) SPF mice were bought from Chengdu Dossy Experimental Animals Co., Ltd. [License number SCXK (Sichuan) 2014-26]. Males (average weight 180–220 g) SPF Sprague-Dawley (SD) rats were purchased from Chengdu Dossy Experimental Animals Co., Ltd. [License number SCXK (Sichuan) 201509]. Both of them were kept in well ventilated sterile polypropylene cages in the animal houses of Sichuan Agricultural University (Chengdu, China). Each cage contained equal number of mice or rats of the same sex. Based on the Guidelines of the International Committee on Laboratory Animals, they were maintained at a controlled temperature of 25 ± 3°C and relative humidity of 55 ± 5% and 12 h light/dark cycle with the lights off at 7 p.m. They were treated with a started diet from Nuvital Nutrients (Colombo, PR, Brazil) and allowed access to sterilized water. Experiments were started after the animals acclimating for a week.

### 2.3. Ethics Statement

All procedures involving animals and their care in this study were approved by the Ethics Committee of Sichuan Agricultural University according to The Regulation of Experimental Animal Management (State Scientific and Technological Commission of the People's Republic of China, number 2, 1988) and The Interim Measures of Sichuan Province Experimental Animal Management (Science and Technology Bureau of Sichuan, China, number 25, 2013).

### 2.4. Analgesic Assay

#### 2.4.1. Acetic Acid-Induced Abdominal Writhing Test

Fifty mice were randomly divided into five groups (containing an equal number of both males and females). The mice were treated with physiological saline, indomethacin (2 mg/kg), and high (30 mg/kg), medium (10 mg/kg), and low (3 mg/kg) dose of RSV, respectively. The mice were orally treated once a day for 4 days. On the 4th day, after administrated for 1 h, the mice in each group were intraperitoneally injected with 0.7% AC (10 mL/kg) [14]. Then, the number of writhes within 20 min (writhe reactions including abdominal contractions, stretching of hind paws, writhing of abdominal muscles, and times of hips up) was recorded [2]. The analgesic percentage was calculated as follows:

(1) Inhibition  rate%=number  of  writhes  control−number  of  writhes  treatednumber  of  writhes  control×100%.

#### 2.4.2. Hot Plate Test

The female mice were staying on a hot plate with a constant temperature (55.5 ± 0.5°C). The activities, including lifting, licking hind paws, and even jumping were considered as the antinociceptive indicators [15]. The female mice with a pain threshold (the time which the mice needed when they first exhibit one of the antinociceptive indicators on the hot plate) within 5~30 s were qualified for the test. Fifty qualified mice were randomly divided into five groups; they were treated with physiological saline, indomethacin (2 mg/kg), and RSV (30, 10, and 3 mg/kg), respectively. The mice were orally administrated once a day for 4 days. On the 4th day, after the orally administration, the pain threshold of mice in each group was determined at 30, 60, 90, and 120 min, respectively. During the test, if the mouse showed no antinociceptive indicator within 60 s, the pain threshold was calculated as 60 s and the mouse was removed immediately.

### 2.5. Anti-Inflammatory Assay

#### 2.5.1. Xylene-Evoked Ear Oedema Test

Fifty mice were randomly divided into five groups of 5 males and 5 females each. Different groups of animals were treated with physiological saline, DXM (2 mg/kg), and RSV (30, 10, and 3 mg/kg), respectively. The mice were orally administrated once a day for successive 7 days. On the 7th day, after administration for 1 h, the mice in each group were daubed with 0.03 mL xylene on the two sides of right ear and the left was used as control [16]. After the application of xylene for 1 h, all mice were euthanized under ether anaesthesia and both ears were cut down along the auricle baseline. The round pieces of ears were taken by a punch (diameter, 8 mm) and weighed by the electronic balance.

(2) The  ear  oedema  degree  A=weight  of  right  piece−weight  of  left  piece,Inhibition  rate%=Difference  in  weight  of  ear  control−Difference  in  weight  of  ear  treatedDifference  in  weight  of  ear  control×100%.

#### 2.5.2. Acetic Acid-Induced Acute Pleurisy Test

Forty-eight male rats were randomly divided into the following six groups: control group (physiological saline), negative control group (physiological saline), DXM (2 mg/kg), and high, medium, and low doses of RSV-treated groups (30, 10, and 3 mg/kg). All rats were orally treated once a day for sustaining 7 days. On the 7th day, after administration for 30 min, the rat in each group was anesthetized with ether and then disinfected with ethanol on the right side of the chest. All rats were injected with 0.2 mL 2% AC except the control group (physiological saline). After being inflamed for 2 h, the rats were treated with corresponding drugs or physiological saline again. After 6 h, all animals were euthanized under ether anaesthesia. The blood was collected from abdominal aorta, the serum was separated for the measurement of SOD and NO (SOD was tested by the way of WST-1; NO was measured by the way of nitrate reductive enzymatic). The pleural cavity exudate was collected and its volume was measured. The exudate (100 *μ*L) was used for WBC count by cell counting chamber (Shanghai Chemical Equipment Co., Ltd.).

#### 2.5.3. Carrageenan-Induced Acute Air-Pouch Synovitis Test

Forty-eight male rats were randomly divided into the following six groups: control group (physiological saline), negative control group (physiological saline), DXM (2 mg/kg), and high, medium, and low doses of RSV-treated groups (30, 10, and 3 mg/kg). On the first day of treatment, each rat was subcutaneously injected with 10 mL filtered air (0.22 *μ*m filter) in scapular area of the back. On the third and sixth days, each rat was injected with 5 mL filtered air in the same way. On the 6th day, if there were no redness, induration, and obvious inflammation reactions on rat's scapular area, we considered that the air-pouch synovitis model was successfully made [17]. On the 7th day, after the administration, all rats were injected with carrageenan (25 mg/kg) in their air-pouch except the control group (physiological saline). At 12 h after the injection, all rats were anesthetized with ether and the blood was collected from abdominal aorta. Then, the serum was separated for the measurement of MDA and T-SOD (MDA was tested by the way of TBA; T-SOD was measured by hydroxylamine method). The air-pouch of each rat was lavaged by 4 mL ice physiological saline containing 50 U/mL heparin, the lavage exudate was collected, and its volume was measured. The lavage exudate (100 *μ*L) was used for WBC count; the rest was separated for the determination of TP, PGE_2_, NO, and MDA.

### 2.6. Statistical Analysis

Means and standard deviations were calculated. The statistical significance (*P* < 0.05 and *P* < 0.01) was compared among the control and experimental groups by using SPSS 17.0 software analysis of variance (ANOVA) followed by the Student-Newman-Keuls test.

## 3. Results and Discussion

Due to the presence in virtually all human and animal diseases, inflammation and pain have become the focus of global scientific research, while current analgesics and NSAIDs have several adverse effects during the treatment process [18, 19]. Hence, the medicinal plants have been considered as a source of medicines for therapeutic applications [20]. In present study, several classic animal models were built in order to provide a sufficient proof for analgesic and anti-inflammatory activities of RSV.

It is known that abdominal constriction induced by AC is a nonselective model because it releases endogenous mediators (prostaglandins) which are capable of stimulating the peripheral nociceptor(s) and neurons sensitive to NSAIDs, opioids, and other centrally acting drugs [21]. In our study, we found, compared with the control group, the number of writhes in indomethacin-treated and RSV-treated groups was all significantly reduced (*P* < 0.01), and the inhibition rates in the high, medium, and low doses of RSV-treated groups (22.22%, 50.14%, and 25.35%, resp.) were all lower than that in the indomethacin-treated group (58.12%), as shown in Table 1. It indicated that indomethacin and RSV had a potent analgesic effect and indomethacin showed better activity than RSV. This result was consistent with the study of analgesic activity of RSV in the capsaicin and glutamate models [22].

Hot plate test is useful for the evaluation of centrally acting analgesics and these analgesics are universally used to elevate the pain threshold of mice towards heat [23, 24]. As shown in Table 2, the results showed that, compared with the control group, the pain thresholds in the indomethacin-treated and RSV-treated groups were all significantly increased at different time points (*P* < 0.05), especially at 60 min (*P* < 0.01). This result suggested that RSV could obviously prolong the time standing on hot plate and could improve the heat-resisting ability of the mice, revealing that RSV had effective analgesic activity.

Since it was confirmed that the RSV had potent analgesic action, we also studied the anti-inflammatory effects. Xylene-induced ear oedema is used in the screening of anti-acute inflammatory activity and in the evaluation of anti- inflammatory activity of steroids [25, 26]. In our study, after the application of xylene on both sides of right ear, significant swelling could be seen (Table 3). Compared with the control group, the ear oedema degree of rats in the DXM-treated and RSV-treated groups was significantly inhibited (*P* < 0.01), and the inhibition rate in the DXM-treated group was 40.87% and in the high, medium, and low doses of RSV-treated groups was 17.06%, 24.61%, and 30.15%, respectively. It suggested that RSV and DXM possessed an anti-inflammatory activity and DXM showed better activity than RSV. But this may not be effective in anti-acute inflammatory disorder but rather in acute inflammatory disorder. This result was relevant with the existing finding that RSV could inhibit inflammation at comparable doses [16].

Carrageenan has been widely applied as a noxious agent to induce experimental inflammation for the screening of plant possessing anti-inflammatory activity, and the application of AC-induced pleurisy test is the same. Previous research showed that the mechanism of carrageenan-evoked inflammation is that the external stimulus is devoured by mastocyte that leads to the degranulation of mastocyte and the releasing of inflammatory mediators [27]. Just as the change of WBC number is an important indicator that suggested to inflammation [28], the inflammatory mediators are a kind of chemical medium with a strongly biological activity, which can not only accommodate the release of itself but also activate other media systems that produce a series of cascade amplification reactions and increase the further development of inflammation [29]. In current study, we not only conducted AC-induced pleurisy and carrageenan-evoked acute air-pouch synovitis tests on the basis of the previous researches [30, 31], but also examined the WBC number and inflammatory mediators both in serum and in lavage exudate. And in both AC-induced pleurisy and carrageenan-evoked acute air-pouch synovitis tests, when compared with the control group, there were no significantly differences on the volume of exudate and the number of WBC in treated (DXM and RSV) groups (*P* > 0.05). While compared with the negative control group, RSV and DXM could obviously inhibit the accumulation of exudate and the migration of WBC (*P* < 0.01, shown in Tables 4 and 5), suggesting that RSV and DXM could remarkably resist inflammation reactions caused by AC and carrageenan through inhibiting the inflammation indicator in body exudate.

**Figure 2:**
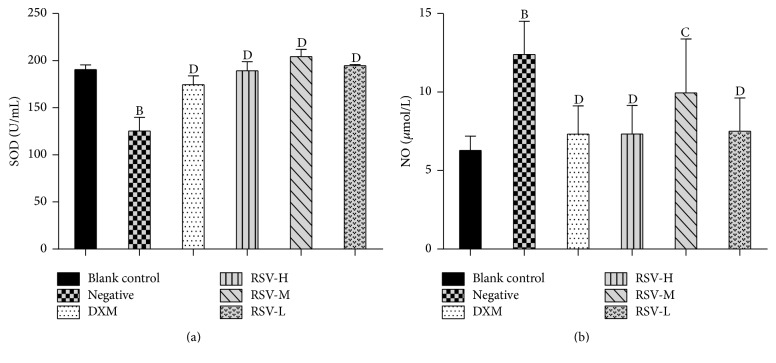
Anti-inflammatory effects of RSV on acute pleurisy test induced by acetic acid. *n* = 8 (*x* ± *s*). DXM, RSV-H, RSV-M, and RSV-L represent the groups treated with DXM, high, middle, and low dose of resveratrol, respectively. (a) The activity of SOD in serum. (b) The content of NO in serum. Mean values followed by different digits indicate significant statistical differences. ^B^*P* < 0.01 versus blank control. ^C^*P* < 0.05; ^D^*P* < 0.01 versus negative control.

**Figure 3:**
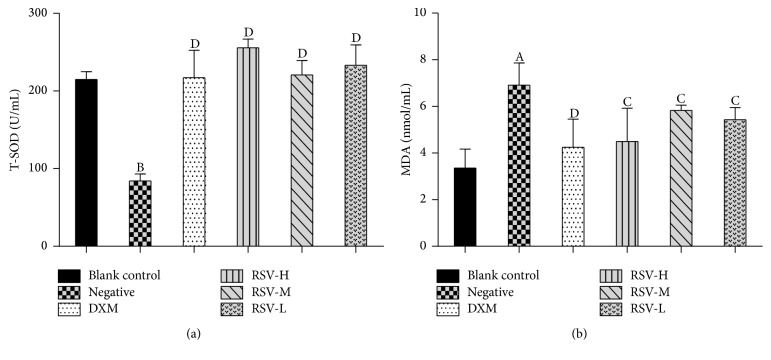
Anti-inflammatory effects of RSV on acute air-pouch synovitis test induced by carrageenan. *n* = 8 (*x* ± *s*). DXM, RSV-H, RSV-M, and RSV-L represent the groups treated with DXM, high, middle, and low dose of resveratrol, respectively. (a) The activity of T-SOD in serum. (b) The content of MDA in serum. Mean values followed by different digits indicate significant statistical differences. ^A^*P* < 0.05; ^B^*P* < 0.01 versus blank control. ^C^*P* < 0.05; ^D^*P* < 0.01 versus negative control.

The serologic parameter is one of the most sensitive parameters to analyze the illness of disease in humans and animals. NO and SOD play an important role in acute and chronic inflammatory processes [32, 33]. As shown in Figure 2, in the AC-evoked pleurisy test, there were no significant differences on the contents of NO and the SOD activities between the control and treated (DXM and RSV) groups (*P* > 0.05), but significant differences were detected in treated (DXM and RSV) groups and negative control group (*P* < 0.01). These results illustrated that DXM and RSV had an inhibitory effect on against pleurisy disease and they could impel the NO and SOD to the normal level. The SOD activities in RSV-treated groups were higher than that in the DXM-treated group, suggesting that RSV is better than DXM on restoring SOD activity, which may attribute to the immunoenhancing and antioxidant activities of RSV. This result confirmed the reports that RSV is a potential therapeutic intervention for aging- or oxidative stress-associated disorder [34]. MDA is one of the end-products of lipid peroxidation [35] and it is also a mediator of sterile inflammation [36]. For carrageenan-induced acute air-pouch synovitis test (Figure 3), there were no significant differences on the contents of MDA and the T-SOD activities between the treated (DXM and RSV) groups and control group (*P* > 0.05), but significant differences were detected when compared with the negative control group. Compared with the negative control group, the MDA contents in serum were obviously decreased in the treated (DXM and RSV) groups (*P* < 0.05), while the T-SOD activities were significantly increased (*P* < 0.01). These results suggested that the DXM and RSV had a treatment effect on acute air-pouch synovitis disease caused by carrageenan and they also could restore the MDA and T-SOD to the standard level. The decrease of MDA in the RSV-treated groups was similar with the previous report that MDA increased in 7,12-dimethylbenz(a)anthracene (DMBA) group but decreased in DMBA group treated with RSV (DMBA + R) [37]. The result is that T-SOD activities in RSV-treated groups were higher than that in DXM-treated group and the result of SOD activity in the AC-evoked pleurisy test was accordantly testified.

**Figure 4:**
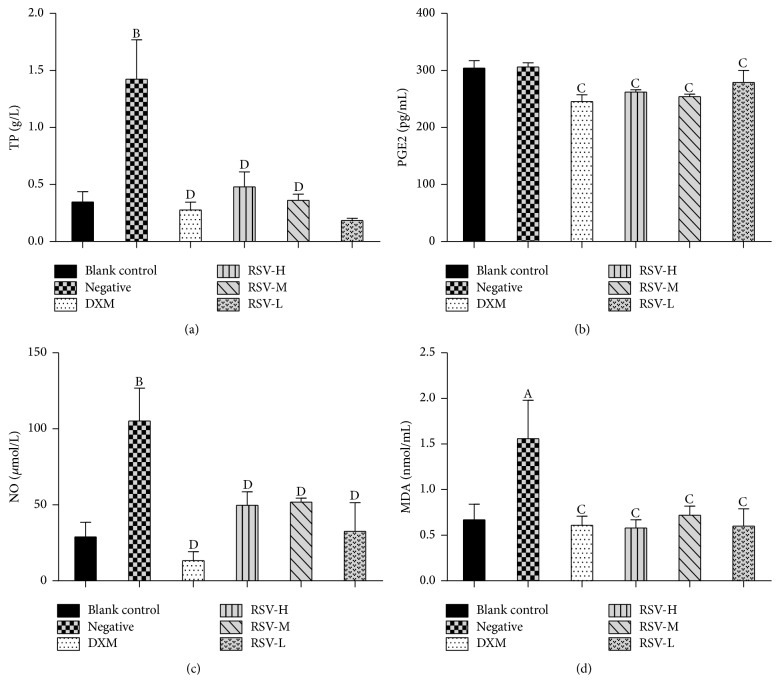
Anti-inflammatory effects of RSV on acute air-pouch synovitis test induced by carrageenan. *n* = 8 (*x* ± *s*). DXM, RSV-H, RSV-M, and RSV-L represent the groups treated with DXM, high, middle, and low dose of resveratrol, respectively. (a) The content of TP in exudate. (b) The content of PGE2 in exudate. (c) The content of NO in exudate. (d) The content of MDA in exudate. Mean values followed by different digits indicate significant statistical differences. ^A^*P* < 0.05; ^B^*P* < 0.01 versus blank control. ^C^*P* < 0.05; ^D^*P* < 0.01 versus negative control.

The examination of lavage exudate could diagnose the degree of inflammation. In carrageenan-induced acute air-pouch synovitis assay, we not only measured the volume and WBC in lavage but also examined the inflammatory mediators in it. Proteins are important biological macromolecules in vital activities; almost all diseases in human are associated with proteins. TP is the sum of all kinds of protein in body; the examination of TP has a huge significance on disease diagnosis [38]. PGE2 is the main metabolite of arachidonic acid and is related to many pathophysiological processes, such as inflammation, tumor, microcirculation of blood, pain, and reproductive. Therefore, the examination of PGE2 is also important for inflammation diagnosis [39]. The results of inflammatory mediators in lavage exudate showed that the contents of TP, NO, and MDA in the negative control group were significantly higher than that in the control group, but PGE2 showed no statistical differences between them (*P* > 0.05) (Figure 4). These results revealed that inflammation reactions happened and inflammatory mediums were activated after injection with carrageenan, but rats in the control group may be influenced by external environment, finally leading to the high expression of PGE2. The results also showed that the contents of TP, NO, PGE2, and MDA in the treated (DXM and RSV) groups were all significantly lower than that in the negative control group, but no statistical differences were observed when compared with the control group (*P* > 0.05). These results suggested that RSV had a remarkable inhibitory effect on the release of inflammatory mediates and may be one of the reasons for anti-inflammatory mechanism of RSV.

## 4. Conclusion

**Figure 5:**
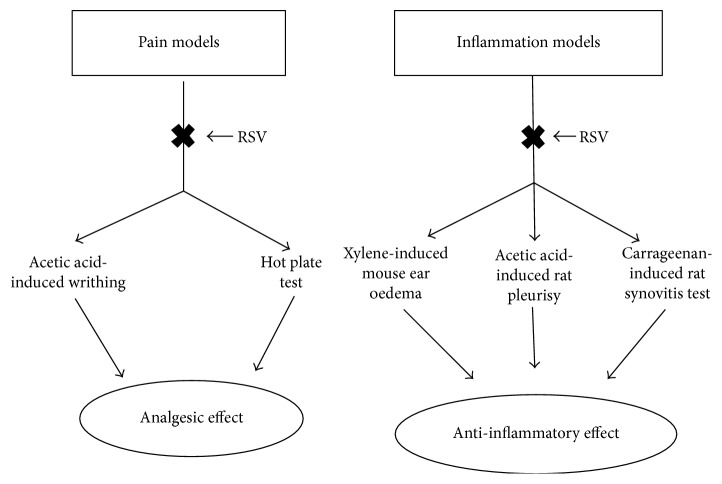
Technical routes of the present experiment. Shortly speaking, RSV possesses analgesic effect and anti-inflammatory effect through inhibiting the pain models and inflammation models in mice and rats.

As shown in Figure 5, in conclusion, RSV inhibited the varieties of pain and inflammation models and exerted a treatment effect on pain and inflammation. The possible mechanism of RSV for its anti-inflammatory effects may be related to regulating the release of inflammatory indicator and inflammatory mediators both in body exudate and in serum. Moreover, RSV has less side effects and can improve the body immunity. Hence, RSV possesses good potential to be used as an adjunctive or alternative therapy for pain and inflammation in the future.

## Figures and Tables

**Table 1:** Analgesic effects of RSV on writhing test induced by acetic acid. *n* = 10 (*x* ± *s*).

Groups	Dosage	Number of writhes	Inhibition rate%
Control	—	58.50 ± 7.74	—
Indomethacin	2 mg/kg	24.50 ± 4.51^b^	58.12
RSV-H	30 mg/kg	45.50 ± 3.62^b^	22.22
RSV-M	10 mg/kg	29.17 ± 6.15^b^	50.14
RSV-L	3 mg/kg	43.67 ± 2.25^b^	25.35

RSV-H, RSV-M, and RSV-L represent the groups treated with high, medium, and low dose of resveratrol, respectively. In each line, different digits indicate significant statistical difference. ^b^*P* < 0.01 versus blank control.

**Table 2:** Analgesic effects of RSV on hot plate test. *n* = 10 (*x* ± *s*).

Groups	Dosage	Pain threshold on different time points			
		30 min	60 min	90 min	120 min
Control	—	6.33 ± 1.16	6.67 ± 1.53	9.33 ± 2.31	10.33 ± 2.52
Indomethacin	2 mg/kg	15.36 ± 2.08^a^	16.67 ± 4.51^b^	14.00 ± 2.65^a^	16.00 ± 5.20^a^
RSV-H	30 mg/kg	14.00 ± 5.29^a^	12.67 ± 1.53^b^	15.67 ± 6.03^a^	13.33 ± 3.06
RSV-M	10 mg/kg	18.33 ± 7.51^a^	12.33 ± 3.21^b^	15.33 ± 2.52^a^	21.67 ± 5.03^b^
RSV-L	3 mg/kg	20.33 ± 6.028^a^	16.67 ± 2.52^b^	22.00 ± 8.72^a^	21.33 ± 2.52^b^

RSV-H, RSV-M, and RSV-L represent the groups treated with high, medium, and low dose of resveratrol, respectively. In each line, different digits indicate significant statistical difference. ^a^*P* < 0.05; ^b^*P* < 0.01 versus blank control.

**Table 3:** Anti-inflammatory effects of RSV on ear oedema test induced by xylene. *n* = 10 (*x* ± *s*).

Groups	Dosage	Ear oedema degree (mg)	Inhibition rate%
Control	—	8.400 ± 0.834	—
DXM	2 mg/kg	4.967 ± 0.761^b^	40.87
RSV-H	30 mg/kg	6.967 ± 0.344^b^	17.06
RSV-M	10 mg/kg	6.333 ± 0.821^b^	24.61
RSV-L	3 mg/kg	5.867 ± 1.174^b^	30.15

RSV-H, RSV-M, and RSV-L represent the groups treated with high, medium, and low dose of resveratrol, respectively. In each line, different digits indicate significant statistical difference. ^b^*P* < 0.01 versus blank control.

**Table 4:** Anti-inflammatory effects of RSV on the number of WBC and the volume of exudate of acute pleurisy test induced by acetic acid. *n* = 8 (*x* ± *s*).

Groups	Dosage	Volume of exudate (mL)	WBC (×10^9^/L)
Control	—	2.533 ± 0.451	3.267 ± 1.201
Negative control	—	3.967 ± 0.153^b^	11.850 ± 2.471^b^
DXM	2 mg/kg	2.333 ± 0.306^d^	5.867 ± 1.266^d^
RSV-H	30 mg/kg	2.633 ± 0.473^d^	5.550 ± 1.053^d^
RSV-M	10 mg/kg	2.533 ± 0.416^d^	6.383 ± 0.775^d^
RSV-L	3 mg/kg	2.033 ± 0.404^d^	5.717 ± 1.144^d^

RSV-H, RSV-M, and RSV-L represent the groups treated with high, medium, and low dose of resveratrol, respectively. In each line, different digits indicate significant statistical difference. ^b^*P* < 0.01 versus blank control. ^d^*P* < 0.01 versus negative control.

**Table 5:** Anti-inflammatory effects of RSV on the number of WBC and the volume of lavage fluid of acute air-pouch synovitis induced by carrageenan. *n* = 8 (*x* ± *s*).

Groups	Dosage	Volume of lavage fluid (mL)	WBC (×10^9^/L)
Control	—	3.233 ± 0.153	66.67 ± 12.50
Negative control	—	4.933 ± 0.153^b^	142.33 ± 6.35^b^
DXM	2 mg/kg	3.533 ± 0.153^d^	44.00 ± 12.77^d^
RSV-H	30 mg/kg	3.200 ± 0.985^d^	86.00 ± 8.89^d^
RSV-M	10 mg/kg	3.400 ± 0.100^d^	85.00 ± 21.17^d^
RSV-L	3 mg/kg	3.400 ± 0.400^d^	72.67 ± 34.59^d^

RSV-H, RSV-M, and RSV-L represent the groups treated with high, medium, and low dose of resveratrol, respectively. In each line, different digits indicate significant statistical difference. ^b^*P* < 0.01 versus blank control. ^d^*P* < 0.01 versus negative control.
